# Descriptive study: the novel “full spectrum people-with-opioid-use-disorder care model”

**DOI:** 10.1186/s12954-023-00778-x

**Published:** 2023-04-12

**Authors:** Richard Kelly, Snehal Bhatt, Jessica Gross, Juan Antonio Dixon, Phillip Fiuty, Max Shapiro, Rafael Fernandez-Mancha, Julie Salvador

**Affiliations:** 1 University of New Mexico, Albuquerque, USA; 2 The Mountain Center, Santa Fe, USA; 3 Santa Fe Preparatory School, Santa Fe, USA

**Keywords:** Harm reduction, Low-barrier, Buprenorphine, Overdose, Opioid use disorder

## Abstract

**Background:**

People with Opioid Use Disorder (PWOUD) represent an underserved and marginalized population for whom treatment gaps exist. Low-barrier programs like mobile care units and street outreach programs have yielded increased access to buprenorphine and social services, however, OUD pertinent co-occurring behavioral health and medical conditions are frequently left unaddressed. A novel, tailored, comprehensive care delivery model may reduce disparities and improve access to care across a range of pathologies in this historically difficult to reach population and enhance efforts to provide universal treatment access in a harm reduction setting.

**Methods:**

Descriptive data were collected and analyzed regarding patient demographics, retention in treatment and services rendered at a new, wrap-around, low-barrier buprenorphine clinic established at an existing harm reduction site in New Mexico between August 1, 2020, and August 31, 2021.

**Results:**

203 people used any service at the newly implemented program, 137 of whom specifically obtained medical and/or behavioral health care services including prescriptions for buprenorphine at least once from the physician onsite. Thirty-seven unique medical and psychiatric conditions were treated, representing a total of 565 separate encounters. The most common service utilized was buprenorphine treatment for opioid use disorder (81%), followed by treatment for post-traumatic stress disorder (62%), anxiety (44.5%) and depression (40.9%). Retention in buprenorphine treatment was 31.2% at 6 months.

**Conclusions:**

An innovative, multidisciplinary, buprenorphine-centric care model, which targets a wide range of OUD pertinent pathologies while employing a harm reduction approach, can enhance utilization of these services among an underserved PWOUD population in a manner which moves our health system toward universal OUD treatment access thereby potentially reducing overdose and existing disparities.

## Introduction

The opioid epidemic represents one of the most devastating public health crises in United States history. Currently in its fourth decade, it reflects few signs of near-term attenuation. Data from the CDC indicate that there were an estimated 100,306 drug overdose deaths in the United States during the 12-month period ending in April 2021, an increase of 28.5% from year before. Estimated overdose deaths from opioids increased to 75,673; up from 56,064 the year before [1]. People with Opioid Use Disorder (PWOUD) represent a population with high need for access to evidence-based drug treatment and health care services. PWOUD face significant health disparities, including a lack of access to the life-saving FDA-approved treatment buprenorphine, which has robust empirical evidence supporting its efficacy, and medical services which are tailored to their specific needs [2–4]. PWOUD are a historically difficult population to reach due to a variety of factors, most notably discrimination, stigma and social marginalization. [5–7]

Prior studies have examined implementation efforts to expand access to buprenorphine in unique settings, such as mobile treatment units and street outreach teams [8–10]. Findings from these studies are promising vis-a-vis improving access to buprenorphine, but these programs remain somewhat siloed in terms of providing access to other types of essential healthcare. This is important because the PWOUD population may benefit from access to low-barrier, comprehensive treatment in which harm reduction [11], trauma-informed care, and evidence-based treatment of substance use disorders are integrated with wrap-around psychosocial support, medical care targeting prevalent conditions in the OUD population, such as hepatitis C, and simultaneous psychiatric care. As part of a federally funded program to address Opioid Use Disorder (OUD) in New Mexico, we developed and implemented the novel “Full Spectrum People With Opioid Use Disorder (PWOUD) Care Model” (referred to as the Full Spectrum program hereafter) providing both OUD treatment and a range of integrated primary and specialized healthcare services. This model is an innovative, far-reaching, multidisciplinary, low-barrier approach designed to address the morbidity and mortality associated with OUD and mitigate the ongoing overdose crisis in rural, underserved Rio Arriba County, NM; which had the 20th highest fatal overdose rate in the country at the time of the project (see Table 1) [12]. The purpose of this study was: (1) to describe utilization of services among PWOUD embedded at the local HRS, and (2) to examine retention in buprenorphine treatment for OUD over time.

**Table 1:** Top 20 US counties for total drug overdose death rate based on age-adjusted rates, 2019

Ranking	County	County code	Deaths	Population	Adjusted age rate
1	Scioto County, OH	39,145	86	75,314	129.4
2	Cabell County, WV	54,011	105	91,945	128.6
3	Washington Parish, LA	22,117	51	46,194	116.1
4	Baltimore city, MD	24,510	731	593,490	114.6
5	Fayette County, IN	18,041	21	23,102	106.8
6	Raleigh County, WV	54,081	69	73,361	103.7
7	Logan County, WV	54,045	31	32,019	103.4
8	Kanawha County, WV	54,039	149	178,124	90.4
9	Fayette County, WV	54,019	31	42,406	88.2
10	Gallia County, OH	39,053	23	29,898	87.0
11	Hancock County, WV	54,029	22	28,810	86.1
12	Wayne County, IN	18,177	49	65,884	83.3
13	St. Louis city, MO	29,510	252	300,576	80.7
14	Salem County, NJ	34,033	42	62,385	80.0
15	Greenup County, KY	21,089	27	35,098	77.0
16	Cecil County, MD	24,015	74	102,855	76.0
17	Cheatham County, TN	47,021	28	40,667	71.5
18	Wayne County, WV	54,099	27	39,402	70.6
19	Cape May County, NJ	34,009	52	92,039	68.8
20	Rio Arriba County, NM	35,039	25	38,921	67.6

Source: CDC Wonder Online Database

## Methods

### Intervention design

We designed the clinic according to a low-barrier model (aka low-threshold model), in parallel with the framework by Jakubowski et al. [13] (see glossary) The Full Spectrum program was implemented via an agency named The Mountain Center at their Harm Reduction Site (HRS) in Espanola, NM, which already offered services such as syringe exchange, psychotherapy, and case management amongst other harm reduction care for PWOUD (see glossary). The project was implemented in collaboration with the University of New Mexico (UNM) Department of Psychiatry and Behavioral Sciences (DPBS). The objective of the Full Spectrum program was to integrate these new services into the existing agency’s service array so they could be more easily accessed by PWOUD in a setting where they were already receiving care. The Full Spectrum program includes medical, addiction, and mental health services tailored to the specific needs of the PWOUD population. Specifically, prescribing services added to the existing services at The Mountain Center include medication for opioid use disorder (MOUD) e.g., buprenorphine primarily, HIV screening and treatment, HCV screening and treatment, STI screening and treatment, Pre Exposure Prophylaxis (PreP), Post Exposure Prophylaxis (PEP), pregnancy screening, birth control, and basic primary care services such as non-insulin dependent diabetes management and hypertension management. These services were paired with collaboration and referral to the local Federally Qualified Health center (FQHC) if the patient needed a higher level of care. The integrated addiction, medical and psychiatric prescribing services were provided by a faculty physician from the UNM DPBS, with back up from the Chief of the UNM addiction psychiatry division, and an LPN and a medical assistant/care coordinator that were provided by The Mountain Center. This core team was also supported by the Executive Director of the Mountain Center as well as their Clinical Director, counselors, a case manager and the harm reduction Program Manager and staff.

### Participant recruitment

Individuals with OUD who visited the HRS were made aware of the services being offered through the full-spectrum program. If the client was interested, they had the option to meet with the physician to receive medication management for their psychiatric and/or somatic pathologies, and to access the other services being provided on-site as part of the program, such as psychotherapy, case management and care navigation.

### Data collection

Data were collected from August 1st, 2020, to August 31st, 2021, using REDCap HIPAA compliant data collection system. Data collected included patient demographics and services utilization. Data was gathered and entered by staff at the Mountain Center, with monthly review of the database with the lead author to ensure completeness and accuracy. The UNM IRB approved this study. Data was downloaded into an excel file for analysis that was conducted by the UNM CTSC (co-author).

### Analysis

We completed descriptive analysis to elucidate the characteristics of the clients in the program and show retention in care at multiple timepoints. We evaluated retention in care at approximately 1, 3 and 6 months after the initial visit, with follow up timing tolerances consistent with O’Guerk et al. [8]. Individuals enrolled throughout the data collection period, therefore, not all patients were in the study for a full six months. We calculated retention based on the number of patients that were in the study for the given time interval. For example, patients who enrolled in the study less than six months before the end of the data collection period were not included in the 6-month retention calculation. Finally, we also tabulated percentages of patients who were prescribed medication for the treatment of specific diagnoses via retrospective manual chart audit.

## Results

### Participation and treatment

**Fig. 1:**
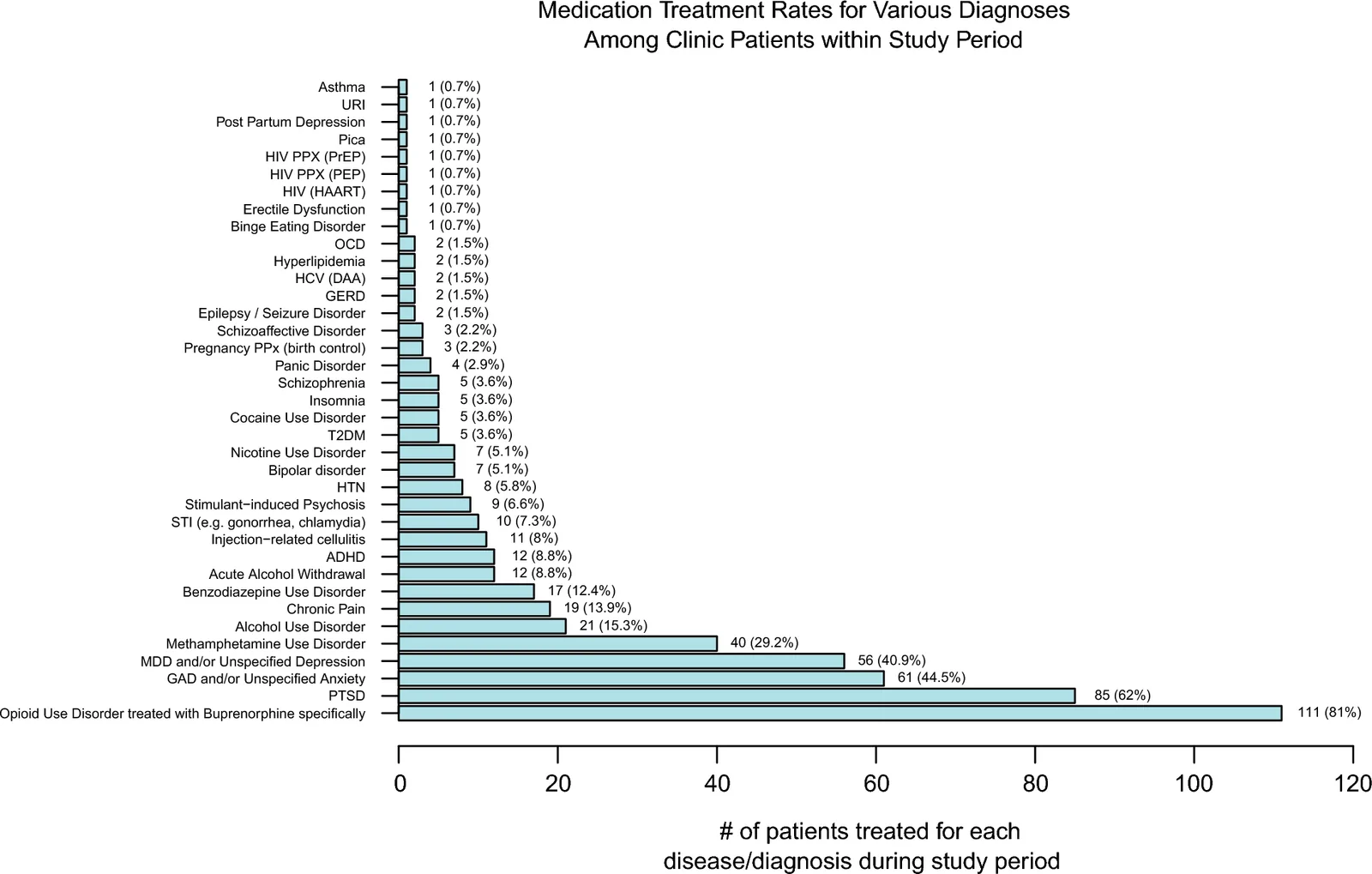
*n* = 37

203 people used services at the low barrier clinic and were therefore eligible for participation in the study. Persons who used services were between the ages of 25–44 (68.5%), white (89.2%), and Hispanic (70.9%) (see Table 2). Of these 203 people, a subset of 137 (67.5%) met with the provider at least once during the study period and were treated for a range of physical and behavioral health diagnoses that were not mutually exclusive (see Fig. 1). A wide array of both psychiatric and somatic pathologies were addressed via medication. Of note, as per the DSM-5, we consider OUD to be a psychiatric disorder and, accordingly, buprenorphine to be a evidence-based psychiatric medication for the purposes of our study. Figure 1 shows that of the 137 people who met with the provider OUD was the most common disorder treated (81%). After OUD, PTSD was the leading co-occurring mental health disorder treated in 62% of patients, followed by Anxiety and Depression at 44.5% and 40.9%, respectively. Potentially severe and persistent mental illnesses such as Bipolar Disorder (5.1%), Schizophrenia (3.6%), and Schizoaffective Disorder (2.2%) were also actively and exclusively managed at the Full Spectrum program. The treatment of patients with concurrent substance use disorders involving methamphetamine (29.2%), alcohol (15.3%) and benzodiazepines (12.4%) also occurred. Notably, infectious diseases related to intravenous drug use (IVDU) were treated and targeted prophylactically, specifically with respect to HCV and HIV. Figure 1 details all provider facilitated medication treatments at the clinic.

**Table 2:** Demographics of people who used low barrier clinic (*n* = 203)

Demographic feature	*n*	Percent of sample (%)
Age range		
18–24	27	13.3
25–44	139	68.5
45–64	37	18.2
Race		
American Indian/Alaskan Indian	16	7.9
Black or African American	4	2.0
More than one race	1	0.49
Native Hawaiian/Other Pacific Islander	1	0.49
White	181	89.2
Hispanic or Latino?		
No	59	29.1
Yes	144	70.9
Is patient pregnant?		
No	202	99.5
Yes	1	0.5
Total sample size	203	100.0

### Retention in treatment

As noted, 137 people obtained medication treatment via the provider embedded with the Full Spectrum program at the HRS (Fig. 1). This represented 565 encounters with the mean number of visits per patient being approximately 4 (4.12). Fifty-six (40.88%) patients visited the clinic only once, 81 (59.12%) patients returned for at least one visit, and the greatest number of visits for a single patient was 52. 364 encounters involved a prescription for buprenorphine, and 43 patients visited the syringe exchange for a buprenorphine prescription a single time. Of the 137 patients, 104 had received buprenorphine medication at least one month before the end of the study period, 85 patients received medication at least three months before the end of the study, and 61 had at least six months before the end of the study. At month one, 33.65% of patients were retained (35/104); at month three 29.41% of patients were retained (25/85); and at month six, 31.15% were retained (19/61).

## Discussion

This study indicates that the novel “Full Spectrum PWOUD Care Model” resulted in patients engaging in both buprenorphine treatment as well as treatment for a variety of other co-occurring disorders. Our buprenorphine retention rate in care at 6 months of 31.15% is comparable to other multidisciplinary programs in an underserved setting (27.6% at 5 months) [8]. Our retention rate at 6 months is also similar to retention rates at open-access treatment settings for example in the Bay Area (27% at 6 months) [9]. Notably, our retention rates are most closely equivalent to care integrated into harm reduction agencies such as in New York City (31% at 6 months) [10]. This finding may be due to the fact that harm reduction agencies foster an environment of safety and trust amongst this traditionally difficult to reach and marginalized population. Staff at harm reduction agencies, who often have lived experience related to Substance Use Disorders (SUDs) themselves, are trained to “meet people where they are at”, non-judgmentally, in a low-pressure manner which fosters trust and engenders a sense of community for PWOUD. We therefore consider the fact that our clinic model was embedded within an existing HRS to be critical to its success. The fact that our clinic provided such a robust, wrap-around, comprehensive menu of services for our clients with a low-barrier approach likely contributed to the solid retention rates as well. Despite relatively robust retention rates in our study in comparison with other similar programs, 31% or less overall is ultimately a suboptimal public health outcome on a population level. This may be due to the high prevalence of psychosocial instability, co-occurring mental health disorders, and deleterious social determinants of health in addition to persistent stigma and discrimination faced by the OUD population. Some patients lost to follow up may have returned to use and face the risk of overdosing fatally. In light of the age of our patients, the vast majority of whom were under the age of 44, this represents tremendous Years of Potential Life Lost.

Furthermore, PTSD was extremely prevalent in our population, a finding consistent with other studies [14, 15]. Our population’s PTSD rates were closely followed by significant prevalence of anxiety and depression. Research shows that treatment outcomes are optimized when both substance use disorders and other psychiatric illnesses are treated in parallel [16]. Our study findings, combined with research evidence, clearly emphasize the need for simultaneous treatment of both OUD and concurrent mental health disorders preferably by the same provider or at the same clinic site. The tendency in our mental health system toward siloed addiction and mental health treatment is inadequate. Furthermore, while we were able to provide prescriptions for buprenorphine, the clinic was not set up to provide buprenorphine directly. Given national pharmacy-level challenges in filling of buprenorphine prescriptions [17], having a pharmacy on site would be an additional advancement benefiting persons with OUD.

Strengths of the program implementation included having the partnership of The Mountain Center as an existing HRS. As noted, being able to leverage the significant psychosocial and harm reduction services they already had in place and their strong reputation and trust among the PWOUD community in Rio Arriba County, NM was paramount. Another strength of the program included the fact that we tried to make access to care as simple as possible, which is consistent with the overall low-barrier and harm reduction ethos. Specifically, even if a patient did not have an immediate interest in medication management, we welcomed any client with a tacit diagnosis of OUD to enroll and meet the case manager and physician, thereby allowing them to have access to the full spectrum menu of services. The physician was also able to then screen for various pathologies and do motivational interviewing to explore the client’s ambivalence around MOUD and provide supportive therapy and psychoeducation. The physician was Board Certified in Preventive Medicine with extensive clinical training in both addiction psychiatry and family medicine which helped facilitate the breadth of medical services offered. In addition, the consultation support of UNM Project ECHO with respect to the infectious disease services offered was vital.

Our program adds to the growing body of literature that supports the idea that person-centered, trauma-informed care integrating a wide range of services, can lead to improved outcomes, especially in populations that are traditionally considered difficult to engage in treatment [8–10, 18–21]. This body of literature strongly supports the idea that harm reduction services and treatment services are compatible, exist along a spectrum, and in fact, can complement each other. It is well studied, for example, that many patients receiving treatment for OUD continue to use illicit substances. Similarly, many patients in syringe exchange programs are interested in receiving treatment for OUD [21]. It is also well-studied that patients with OUD are impacted by higher than average rates of psychiatric illnesses [22], medical conditions such as hepatitis C [23], as well as psychosocial difficulties such as unemployment and unstable housing [21] that can profoundly impact an individual’s life. Integrated care can provide a unique opportunity to successfully meet these varied needs in a timely and convenient manner, and, by doing so, improving outcomes for individuals with substance use disorders. Unfortunately, such integrated care remains relatively uncommon. Our program supports the idea of creating integrated, low barrier and flexible systems of care with co-located harm reduction, social, medical, psychiatric, and substance use treatment services. Of note, to our knowledge, no other publication incorporating buprenorphine treatment within unique settings has reported integrated treatment of co-occurring psychiatric illnesses. Here, we demonstrate that this can be achieved, and should be seen as a standard element of integrated care given the high proportion of patients who sought psychiatric care in our program.

Challenges to program implementation included the inherent difficulty of establishing a medical clinic in a non-medical setting. In retrospect, a team member with extensive experience in building medical clinics from the ground up and optimizing clinical workflows, such as a senior clinical nurse manager working on-site, could have been beneficial. Another challenge was administrative/clerical burdens that took away from all staff being able to work at the top of their licensure. It is important whenever possible to adjust staff roles and responsibilities such that all members of the team are operating and practicing at the top of their skill-set in order to minimize burnout and secondhand trauma [24] If not possible, having clear discussions at the outset about the job duties and expectations of staff particularly in the early stages of development of a full spectrum program is imperative. Finally, the program was implemented during the height of the COVID pandemic which involved unprecedented operational hurdles.

## Limitations and future directions

Our study did not attempt to locate patients who did not return for services to understand reasons for not further engaging in care at the low-barrier clinic. Therefore, it is unknown if they obtained care at another facility, relocated to another community, or had other reasons for discontinuation. Additionally, this study was purely descriptive in terms of design and analysis. Therefore, we do not know how demographic characteristics, including diagnoses, may be related to ongoing engagement in services over time.

Future research is needed to mitigate the pernicious effects of OUD and the ongoing overdose crisis. In addition to making advancements in public health and clinical strategies, research gaps and translational opportunities include investigating basic science foundations for efficacious interventions, new medications and better provider training [25]. This should be coupled with aggressive OUD treatment workforce development and retention, primary prevention, utilization of peer-support, and patient/public education focused on stigma reduction. On the other hand, we must resist the temptation to view OUD solely through the lens of mental health pathology thereby medicalizing a phenomenon which is itself the symptom of socioeconomic decay and governmental policy paralysis. Addressing the structural causes of the opioid epidemic, such as a poorly functioning mental health system, poverty, racial injustice, and housing affordability, are ongoing challenges in the United States. In addition to making MOUD as widely available as possible including in jails and prisons, public health approaches such as the decriminalization of the individual possession of small amounts of *all* drugs thereby diverting people with SUDs into voluntary treatment rather than incarceration—as has been done in Portugal and Oregon—are also promising policy strategies [26–29]. Methadone should be deregulated to increase ease of access, naloxone should be distributed aggressively and widely to help reverse overdoses as they are occurring, and Overdose Prevention Centers (OPCs) should be considered viable tools at our disposal to stem the tide of the overdose crisis and funded accordingly (see glossary). Ultimately, the overarching focus must be on humane evidence-based services including harm reduction, rather than shunting people who are suffering toward the criminal justice system and exacerbating mass incarceration in the setting of four decades of rising drug overdose deaths. [30, 31]

## Conclusions

Our study indicates that the novel “Full Spectrum PWOUD Care Model” resulted in some patients engaging in treatment for OUD and other co-occurring physical and behavioral health disorders. Expanding available services at sites like The Mountain Center may be an important step in shifting OUD care toward universal treatment access. The harm reduction approach at our program and a non-stigmatizing environment are likely to be important conditions for PWOUD to feel more open to engaging in other offered services. While not all locations may have the capacity to expand services as fully as were done in our clinic setting, given the site’s ability to utilize specialized physicians, we do believe that even gradual expansion of services in other settings serving PWOUD that include MOUD as well as services for co-occurring disorders, may be particularly helpful for attenuating OUD morbidity and mortality.

## Data Availability

Data are not available for outside review and/or analysis.

## Glossary

Harm reduction/Harm Reduction Site (HRS): A harm reduction site is where the philosophy of harm reduction itself is performed i.e., public health practices aimed at lessening social and health-related consequences for both individuals and communities of people who use drugs; including safer use, managed use and abstinence. These sites historically have often included syringe exchange programs among other social services. The harm reduction ethos in general involves a variety of approaches which include meeting people who use drugs “where they’re at” and without judgement, while simultaneously addressing conditions of use along with the use itself. [11]
Low-barrier/Low-threshold: Describes approaches that attempt to remove barriers to OUD medication treatment. Often guided by principles of: (1) same day treatment entry; (2) harm reduction approach; (3) flexibility; (4) wide availability in places where people with opioid use disorder go. [13]
Overdose Prevention Center (OPC): Aka Supervised Consumption Sites provide spaces for people to inject previously obtained illicit substances with sterile equipment, in settings where they can be observed and others can quickly intervene in the event of an overdose. Generally, OPCs are staffed by experiential (people with lived experience of drug use) and non-experiential harm reduction workers [32]. They remain controversial if not illegal in the United States.
